# Communication and relationship dynamics in surgical teams in the operating room: an ethnographic study

**DOI:** 10.1186/s12913-019-4362-0

**Published:** 2019-07-29

**Authors:** Birgitte Tørring, Jody Hoffer Gittell, Mogens Laursen, Bodil Steen Rasmussen, Erik Elgaard Sørensen

**Affiliations:** 1 Department of Clinical Medicine, Aalborg University, Aalborg, Denmark; 2 Act2learn HEALTH, Aalborg University College Northern Denmark, Aalborg, Denmark; 3 The Heller School for Social Policy and Management, Brandeis University, Waltham, MA USA; 4 Department of Orthopedic, Aalborg University Hospital, Aalborg, Denmark; 5 Department of Anesthesiology, Aalborg University Hospital, Aalborg, Denmark; 6 Clinical Nursing Research Unit, Aalborg University Hospital, Aalborg, Denmark

**Keywords:** Ethnography, Relational coordination, Teamwork, Communication, Relationship, Interdisciplinary, Perioperative nursing, Patient safety

## Abstract

**Background:**

In surgical teams, health professionals are highly interdependent and work under time pressure. It is of particular importance that teamwork is well-functioning in order to achieve quality treatment and patient safety. Relational coordination, defined as “communicating and relating for the purpose of task integration,” has been found to contribute to quality treatment and patient safety. Relational coordination has also been found to contribute to psychological safety and the ability to learn from mistakes. Although extensive research has been carried out regarding relational coordination in many contexts including surgery, no study has explored how relational coordination works at the micro level. The purpose of this study was to explore communication and relationship dynamics in interdisciplinary surgical teams at the micro level in contexts of variable complexity using the theory of relational coordination.

**Methods:**

An ethnographic study was conducted involving participant observations of 39 surgical teams and 15 semi-structured interviews during a 10-month period in 2014 in 2 orthopedic operating units in a university hospital in Denmark. A deductively directed content analysis was carried out based on the theory of relational coordination.

**Results:**

Four different types of collaboration in interdisciplinary surgical teams in contexts of variable complexity were identified representing different communication and relationship patterns: 1) p*roactive and intuitive communication*, 2) s*ilent and ordinary communication*, 3) *inattentive and ambiguous communication*, 4) *contradictory and highly dynamic communication.* The findings suggest a connection between communication and relationship dynamics in surgical teams and the level of complexity of the surgical procedures performed.

**Conclusion:**

The findings complement previous research on interdisciplinary teamwork in surgical teams and contribute to the theory of relational coordination. The findings offer a new typology of teams that goes beyond weak or strong relational coordination to capture four distinct patterns of relational coordination. In particular, the study highlights the central role of mutual respect and presents proposals for improving relational coordination in surgical teams.

## Background

Interdisciplinary collaboration in surgical teams has been extensively studied for years due to concerns regarding the impact of human factors on patient safety in the surgical context. Of particular interest has been how the quality and efficiency of surgical procedures are affected by communication failure [1], and how attitudes concerning medical errors and teamwork influence the quality and efficiency of surgical procedures [1–3]. The quality and efficiency of surgical procedures and patient safety are contingent on high quality communication and shared knowledge, which are challenging to achieve due to the interdependence, time constraints, and uncertainty of the surgical context [4]. Surgical team members need not only clinical knowledge and technical skills. They also need skills to engage in teamwork, to understand the complexity of the clinical situation, to make appropriate decisions and to act efficiently [3, 5–7].

These so-called non-technical skills may be assessed and potentially strengthened through the use of various behavioral measurement systems. These rating systems contain behavioral markers for assessing the presence of the following skills displayed through the health professional’s behavior: situation awareness, decision making, communication and teamwork, task management and leadership [3, 8–11]. These measurement systems may not be sufficient, however.

Evidence-based team training concepts are used in many hospitals to train health professionals and improve surgical teamwork. Implementation of these programs improves communication and interdisciplinary collaboration in the operating room [12] and increases awareness of the importance of human factors on patient safety [13]. Moreover, systematic and continuous team training has a positive effect by reducing mortality and morbidity [14] But the implementation of these programs often encounters multiple barriers, therefore there is a need to better understand how to successfully implement evidence-based practices such as these team training programs [15].

The need to develop a more patient or person-oriented approach to patients requiring surgical treatment has been highlighted. An ethnographic study focusing on operating room nursing underlines the significance of the operating room nurses´ skills to strike the right balance between technical skills and care skills, which requires the dual presence of technical flair and seeing patients as human beings [16]. Clearly, the quality of surgical teamwork is not just a question of teaching surgical team members non-technical skills and learning new management practices. In addition, surgical members need to discuss the plan and establish a shared mental model [17, 18] of what needs to be done during surgery in order to coordinate their work and develop adaptive coordination strategies - especially in challenging moments or unexpected situations [19]. Most surgical teams are established ad hoc, comprised by different team members from day to day. These conditions challenge the team’s adaptive capacity [20] and the interactive dynamics among team members [21]. Lack of knowledge about one another increases the likelihood of miscommunication and interruption during surgical procedures [22]. To avoid such disruptions, team coordination and leadership are needed, especially given that team members must continually switch their focus of attention between the execution of their individual assignments and coordination with the team [23]. The quality of surgical team collaboration is hence rooted in team members´ knowledge and skills in relation to procedures, knowledge of their own and other team members’ roles, and communication processes that support the appropriate modalities of collaboration, notably so in the face of unexpected surgical challenges.

The theory of relational coordination captures many of these insights. Relational coordination is a mutually reinforcing process of communicating and relating across areas of expertise for the purpose of task integration [24]. Relational coordination is comprised of shared goals, shared knowledge, and mutual respect, supported by frequent, timely, accurate, and problem-solving rather than blaming communication. It is a high bandwidth form of coordination that is expected to impact performance most significantly under conditions of task interdependence, uncertainties, and time constraints. Relational coordination has been found to predict higher levels of quality, efficiency, and job satisfaction [25] as well as work engagement [26], psychological safety and the ability to learn from errors [26, 27]. Relational coordination is also related to surgical outcomes such as lower postoperative pain, higher postoperative functioning, and shorter lengths of stay [4]. Furthermore, relational coordination predicts a lower occurrence of hospital related infections, patients´ complaints, and medication errors [28]. Based on this background, relational coordination seems relevant to the functioning of surgical teams in operating rooms. Although extensive research has been carried out regarding relational coordination in many contexts including surgery, no study has explored how relational coordination works at the micro level and which alternative patterns of relational coordination can be found at that level. This study explores the communication and relationships in interdisciplinary surgical teams at the micro level in contexts of variable complexity using the theory of relational coordination.

## Methods

This ethnographic study was based on participant observations and interviews inspired by practical ethnographic principles [29, 30]. Members of interdisciplinary surgical teams were observed and interviewed in their daily tasks performing knee and hip replacement surgery in the operating room. Most surgeries were open, and patients were either in general (16 patients) or regional anesthesia (23 patients). The surgical procedures lasted between 30 to 340 min (average 132 min). Observations were focused on these selected surgical specialties to enable identification of habits and patterns across situations arising in connection with performing comparable surgical procedures of varying complexity.

The teams were set in the beginning of the day by the nurse managers and they included 1–2 surgeons (SG), 1 surgical assistant (SA), 1 surgical nurse (SN), 1 circulating nurse (CN), 2 anesthetic nurses (AN nurse), 1 anesthesiologist (AN) and sometimes 1 nurse assistant (NA). The participants comprised a total of 39 surgical teams including 85 team members. Varying complexity was ensured by recruiting teams from two geographically different units working at different levels of specialization but within the same organizational setting: one regional hospital at which surgery was performed in conformity with fixed care pathways and one university hospital where non-standardized surgery was performed on vulnerable/critical patients (ASA group≥2). The ASA score is a physical status classification system developed by the American Society of Anesthesiologists (ASA), to simply categorize the patient’s physiological status in order to help in predicting operative risk.

The first author (BT) was engaged in passive participation, in the sense of being present at the scene of action in the operation room without participating in the surgical procedures ([30], pp. 58–59). First the observations were conducted from an unstructured investigative approach - *grand tour observations* of dimensions such as: space, actor, activity, object, act, event, time, goal and feeling [30]. These observations were followed by more focused observations with increased awareness of the communication and coordination of the interdisciplinary collaboration *- mini tour observations* [30]. Field notes containing participants´ reports and essential verbal exchanges between participants were written into coherent text by the first author immediately after the daily observations [30, 31]. In order to gain insight into the intentions and attitudes motivating the participants’ behavior, individual semi-structured interviews (in total 15) with operating room nurses, AN nurses, surgeons, and anesthesiologists were carried out on the basis of the previous observations ([32] pp. 120–129). Finally, two semi-structured interdisciplinary group interviews (4–5 participants including SN/CN, AN nurses, SG, AN) were completed to comprehend the participants´ attitudes to the culture of teamwork, and to gain insights about their ways of speaking about interdisciplinary collaboration [33]. The audio recordings from the interviews were fully transcribed by the first author (BT).

The study took place over a period of 10 months in 2014 during which 60 surgical procedures were observed, corresponding to 240 h of observation in 30 days. In total, 39 out of the 60 surgical procedures were observed during the focused observation period (23 routine/ 16 complex). This strategy allowed for repetitions of procedures and conversations between teams, actors, and contexts about technical procedures over time and set aside the “tip-of-the-iceberg” assumption ([30] pp. 70–71).

## Theoretical frame and data analysis

A directed content analysis [34] was carried out based on the theory of relational coordination. The analysis process was inspired by Høyer [35] and the metaphor of using theory as a can opener for opening up and identifying the field of study in an analysis. According to the theory of relational coordination, effective task coordination takes place through a relational network among the professionals who are part of the same work process [36]. There may be appropriate as well as inappropriate dynamics of communication and relationships across different workgroups at the same team [25]. Appropriate communication and relationship dynamics are visible when shared goals, shared knowledge and mutual respect create more frequent, accurate, timely and problem-solving communication, which in turn helps to further strengthen shared goals, shared knowledge and mutual respect. Inappropriate communication and relationship dynamics are visible when functional goals, exclusive knowledge and disrespect contribute to infrequent, inaccurate and delayed communication, which in turn reinforces the functional goals, specialized knowledge and disrespect [4]. During the analysis process, the researcher moved continuously and dialogically between the theory of relational coordination and the empirical materials [37].

Fieldnotes and transcriptions from the interviews were organized as verbatim text in the qualitative data analysis software program NVIVO. The text was read in order to gain knowledge about the characteristics of collaboration in interdisciplinary surgical teams.

The analysis code process consists of five steps, guided by the theory of relational coordination. In the *first step*, all instances of interdisciplinary teamwork observed in the operation room were marked in the fieldnotes. In the *second step*, the presence of appropriate and inappropriate dynamics of communication and relationships were coded - as shown in Tables 1 and 2. In one category (Table 1), the presence of *appropriate* **dimensions** was coded: shared goals, shared knowledge, and mutual respect; accurate, timely, and problem-solving communication. (Table 1) In another category (Table 2), the presence of *inappropriate* **dimensions** was coded: functional goals, specialized knowledge and disrespect; inaccurate, delayed, and blaming communication. (Table 2). In the *third step*, the number of codings’ for all dimensions in each of the 39 surgical teams was counted. This results in a number of codings’ for presence of *appropriate* communication and relationships dynamics (+RC) and a number of codings’ for presence of *inappropriate* communication and relationships dynamics (−RC) for each team. Table 3 shows an example of this step presenting codes for a team (Team 27). Account was taken of the duration of surgery. The number of codes in each surgical team was accordingly time-adjusted and set as codes/hour. Thus, a surgical team with 30 codes for (+RC)/hour and 5.6 codes for (−RC)/hour can be presented graphically by two numbers.

**Table 1:** Coding system for the directed content analysis associated with appropriate communication and relationship dynamics

Category 1: Appropriate Communication and Relationship Dynamics	
Shared goal	*The SG asks,* “*How long will it be before you are ready to take the next patients?” The SN responds, “We may as well go on at once, we just need to clean and make over our preparation.” The AN nurse adds, “Also for our part!” The SG answers, “Then it’s a deal, it’s what we do!” [Team16]*
Shared knowledge	*The CN and SN have just realized that the repulsive saw is missing [a specific instrument usually used for that type of surgery]. The saw will be in the OR in 1½ hours at the earliest. The SN and AN nurse are talking together to coordinate the new time perspective. They agree that the SN might clear the situation with the SG. The CN calls the SG and asks,* “*The patient is in OR now, would you please come and mark the hip? But there is an issue, the repulsive saw is missing and will be here about 1½ hours at the earliest. They [AN nurses] would like to perform the spinal anaesthesia now”. They talk on the phone a little. The CN informs the SN and AN nurse and says,* “*He will come now, and he doesn’t care about the saw. We can move on now”. [Team 24]*
Mutual respect	*OR-Nurse 34 and OR-Nurse 36 are preparing the next surgical procedure and talking about how to allocate the day’s work. OR-Nurse 34 says,* “*Shall I take the first [be the surgical nurse], then you can see how I manage, and you can do it yourself afterward?” OR-Nurse 36 answers,* “*Yes, we can do that, but I would like to take the cemented hip. Yesterday, I was the surgical nurse for three “cementless hips.” I need training with the cementing, so I would really like to do that.” OR-Nurse 34 says,* “*Okay, that’s fine. I’ll take the first two and you take the hip and the last patient with the fasciotomy!” OR-Nurse 36 says,* “*Okay!” [Team16]*
Accurate communication	*The AN nurse is reading from a paper – name of the patient, ID number, and type of surgical procedure. She mentions,* “*Ciproxin has been given”. The SG replies, “Yes, superb and no expected surgical implications. Estimated time for the surgical procedure, half an hour!” [Team 16]*
Timely communication	*The SG takes off his gloves, having just finishing the surgical procedure. The CN says,* “*Look at these pictures [X-rays]. It is from the next patient! What did we agree about? What are we going to do?” Then, they talk about which type of hip replacement materials they are going to use for the next patient. They walk together to the closet and look at the different replacement materials and instrument boxes. They make a choice and decide together. [Team 12]*
Problem solving communication	*The SN says* “*Oh, these two, () they* ***don’t*** *fit together!” The CN thinks and says* “*Oh, NO, we have to stop him [the surgeon]. The head [one part of the replacement materials] he has chosen doesn’t fit in”. She knocks on the door to the room, where the AN nurse is preparing the patient for aesthesia and says, “Wait a minute!” Then she calls the surgeon. The CN and SN discuss the size of the replacement materials and what to do now. The CN says* “*He will come, and he is very annoyed that the person who prescribed the operation was so focused on the thighbone part when the patient’s acetabulum is so damaged”. They are talking about which solutions they should go for. The surgeon arrives, and together they discuss the possibilities and decide. “We will continue! Never going down on equipment!” the surgeon exclaims. [Team 29]*

For each dimension the table shows text from the fieldnotes coded for the dimensions associated with appropriate communication and relationship dynamics

**Table 2:** Coding system for the directed content analysis associated with inappropriate communication and relationship dynamics

Category 2: Inappropriate Communication and Relationship Dynamics	
Functional goal	*The SG says, “I will stick to my fundamental views on this case in terms of unpacking. It is important to think about saving money; we just take the stuff into the OR and pack it up if we need it.” The CN replies,* “*Okay, but if it isn’t prepared, you’ll blame me if we need it during the intraoperative phase!” [Team 13]*
Specialized knowledge	*The SN says,* “*If it is surgeon x operating, he would like to have Number 4 [suturing thread] and he would like to have those knife blades!” “Okay, yes,” the CN answers and finds the thread and blades. “He has some whims, I think!” the SN says to her colleague. “I call it ideas,” the CN replies and continues, “In my opinion, you should adapt to the working place – to some degree. I have tried it once, I had been busy and had fetched lots of instruments and placed them in the box because he wanted them there. But he never used them. So, I am finished doing that!” [Team 18]*
Disrespect	*The AN nurses are preparing the patient for anaesthesia. The OR nurses are waiting, and one of them says,* “*These AN nurses are the sharpest. Look at them!” When asked,* “*In what sense, sharpest?” the OR nurse replies,* “*Look at her, look at her rapid movements. She is so rapid and…” She stops talking. The question was repeated,* “*In what sense? The most proficient or?” The OR nurse explains, “No, they are probably very skilled, but they are also very tough. I don’t say anything. You get yelled at if you do something. I am quiet when I am working with them!” [Team 16]*
Inaccurate communication	*A newly employed SN prepares for the surgical procedure and the CN [experienced supervisor] asks,* “*I need to know, should I keep an eye on you?” The SN asks,* “*What exactly do you mean?” The CN replies,* “*I am wondering, how far you are in your training and how much can you manage by yourself? Am I supposed to tell you what to do, or do you know what is going to happen?” The SN answers, “I am so far into my training that I know what to do and I would like to do it myself. But you should know that I perhaps need more time to prepare. You should tell me if I need to do something. I would like to do it myself; it is the best way of learning and training for me!” The CN replies,* “*You have to ask me if you need something.” “Okay, I will do so,” the SN says and continues, “Those articles we are going to use, is it x [hip replacement article]?” The CN answers,* “*I expect it is, I think, but I don’t know, I have never tried it before!” she shrugs and walks away. [Team 9]*
Delayed communication	*The CN says to the SG,* “*Could we talk about the next patient? She is going to have a cementless hip replacement. Do we have what is needed for that surgical procedure?” The SG answers,* “*I haven’t seen the patient, I must do that first!” The CN groans,* “*I am nearly losing my overview, we have so many things going on today!” [Team 12]*
Finger-pointing communication	*The AN nurse enters the OR and says to OR nurse,* “*I am sorry about my reaction before. It wasn’t good. But it is incredible that we had to stop because the INR hasn’t been controlled [INR levels - an essential component in the management of patients receiving blood- thinning treatment]. We have asked for it all day. So annoying! It is not my responsibility! Someone has been asleep, and so here we are!” [Team 31]*

For each dimension the table shows text from the fieldnotes coded for the dimensions associated with inappropriate communication and relationship dynamics

**Table 3:** Codes for communication and relationship dimensions associated with appropriate and inappropriate dynamics for Team 27

Codes for Communication and Relationship Dimensions in *Team 27*			
Dimensions associated with appropriate dynamics	(+RC) *n*	Dimensions associated with inappropriate dynamics	(−RC) *n*
Shared goal	18	Functional goal	1
Shared knowledge	3	Specialized knowledge	0
Mutual respect	16	Disrespect	3
Accurate communication	10	Inaccurate communication	2
Timely communication	23	Delayed communication	6
Problem-solving communication	5	Finger-pointing communication	2
Total (+RC) Codes	75	Total (−RC) Codes	14
(+RC) codes pr. 60 min	30	(-RC) codes pr. 60 min	5.6

Team 27 performed a complex surgical procedure with the duration of a 150 min

**Fig. 1:**
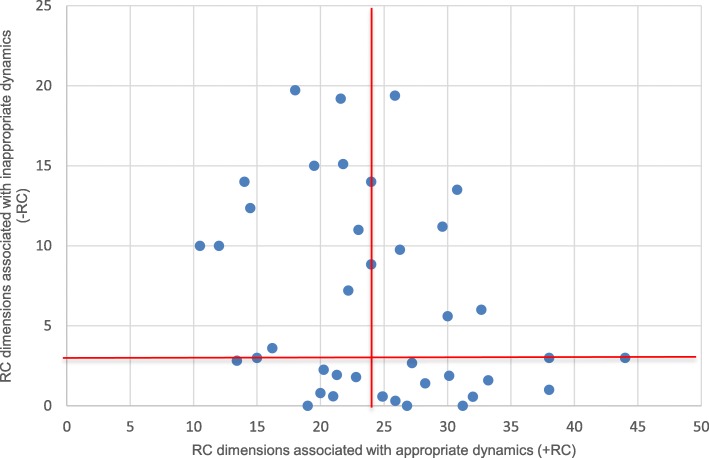
Surgical teams marked by the numbers of codes for communication and relationship dimensions associated with appropriate and inappropriate dynamics. Red lines show the medians (horizontal median = 24, vertical median = 3)

**Fig. 2:**
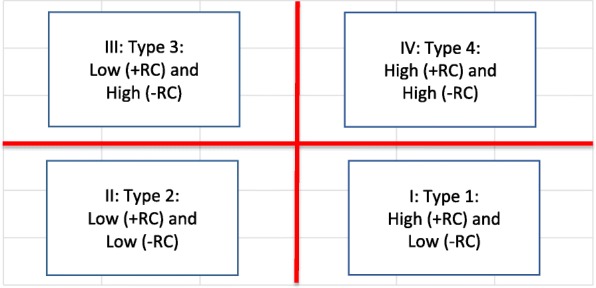
Types of communication and relationship dynamics based on numbers of codes for (+RC) and (−RC)

**Fig. 3:**
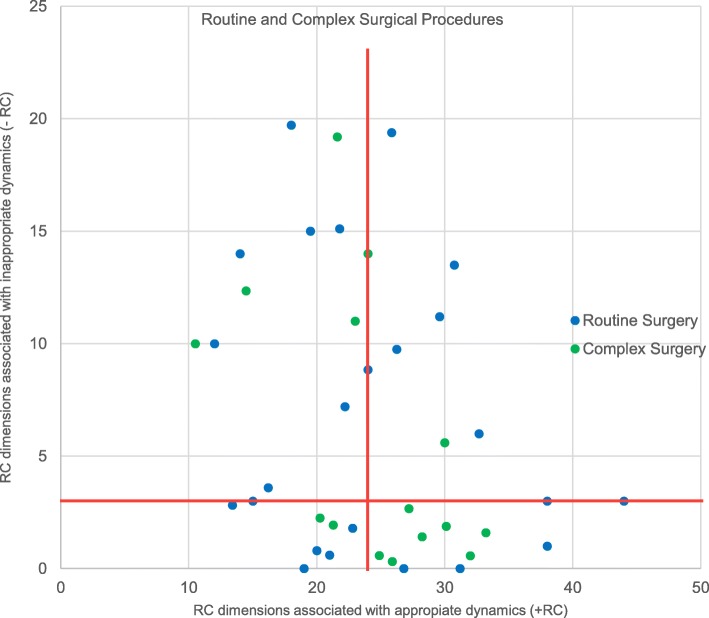
Routine and complex surgical procedures performed by the surgical teams. Routine and complex surgical procedures performed as illustrated in a scatterplot marked by the numbers of codes for appropriate and inappropriate communication and relationship dynamics. Red lines show the medians

In the *fourth step*, surgical teams were illustrated graphically in a matrix where the presence of *appropriate* communication and relationship dimensions (+RC) was marked on a horizontal axis and the occurrence of *inappropriate* communication and relationship dimensions (−RC) was marked on a vertical axis (Fig. 1). Figure 2 presents how medians were marked, and four boxes occurred as an expression of four different types of teamwork: Type 1: High (+RC), Low (−RC); Type 2: Low (+RC), Low (−RC); Type 3: Low (+RC) and High (−RC) and Type 4: High (+RC) and High (−RC). The four types also differed in terms of the level of complexity of the surgical procedures performed (Fig. 3). Figure 3 shows the routine or complex surgical procedures performed. Type 1 included teams that performed the most complex surgical procedures, and the fewest routine surgical procedures. Only three of the 15 complex surgical procedures were performed by Type 2 and Type 4.

An overview of number of coding for all communication and relationship dimensions in the four different types of teamwork is provided in Table 4. *Finally*, data were read again and descriptions of what characterizes the different types of team were prepared.

**Table 4:** Mean of communication and relationship codes in the four different types

Different Communication and Relationship Patterns				
	Mean of codes in Type 1	Mean of codes in Type 2	Mean of codes in Type 3	Mean of codes in Type 4
Shared goal	7.9	4.7	4.7	7.4
Shared knowledge	2.5	2.0	1.3	2.5
Mutual respect	4.5	3.1	2.0	3.5
Accurate communication	5.2	2.6	2.9	4
Timely communication	9.5	5.6	6.3	8.0
Problem-solving communication	2.4	1.2	1.2	2.2
Functional goals	0.1	0.4	2.2	0.9
Specialized knowledge	0.2	0.1	0.5	0.8
Disrespect	0.2	0.4	5.2	5
Inaccurate communication	0.2	0.1	0.7	0.9
Delayed communication	0.4	0.9	3.4	2.2
Finger-pointing communication	0.3	0.1	1.3	1.0

## Results

The following four types represent different ways of communicating and relating seen in daily task performance within surgical teams, based on the directed content analysis:

- Proactive and intuitive communication (Type 1).
- Silent and ordinary communication (Type 2).
- Inattentive and ambiguous communication (Type 3).
- Contradictory and highly dynamic communication (Type 4).

In the following, each citation of an observation or participant quotation has been assigned a reference number for one of 39 surgical teams observed or one of the participants - practicing as an SG, an SN, an CN, a NA, an AN, or an AN nurse.

### Type 1: proactive and intuitive communication

In Type 1 teams, interdisciplinary collaboration was characterized by a broad agreement regarding shared goals, a noticeable expression of mutual respect, and timely and accurate communication focused on solving the problems that arose. Frequently, these teams performed complex surgical procedures of a long duration. The degree of complexity emphasized the need for and the importance of shared responsibility to manage daily surgery schedules in the best possible way. Communication and relationship dynamics in these surgical teams were characterized by participants being proactive and intuitive.

The proactive and intuitive communication was notable when the team members exchanged considerations about expected challenges before and during the surgical procedures, and thus solved problems in advance through shared decision making and problem-solving communication. If unforeseen events occurred (e.g. patients´ conditions, lack of surgical assistants, surgical instruments, or replacement materials needed) and there was a risk that they would cause surgical complications, cancellations, or delays of surgery, team members exchanged their reflections involving all team members expertise and experiences. Together, they searched for the best possible solutions and made appropriate decisions. As demonstrated in the following observation from team activities in the operating room [Team 29]:

*The CN knows exactly which types of materials connect, although it is a very rarely used instrument. She is talking loudly to her colleagues and the surgeon about how and what to do. The SG is listening; he mentions the possible solutions and chooses materials for the replacement of the hip. However, the SG is very concerned about the vulnerable condition of the patient’s bones. “It is quiet thin!”, he says. The AN nurse enters the room and asks if they may sedate the patient. The CN answers: “Yes, we have just made our decisions about the surgical procedure and choice of materials. You may do so!” The AN nurse replies, “Okay, then we will begin sedation of the patient!” The NA works confidently with a rapidly and steady hand during their preparation for the surgical procedure. She talks about her reason for acting and gives the SN much advice. The NA gets the full attention of the other (CN and SN). They are listening and responding to her ideas. The CN nurse prepares the transportable x-rays appliance and says: “We should probably prepare ourselves that it will be done under radiolucency, when the patient's bone tissue is so thin!”*

The participants knew one another’s role and expertise and took into account what was important for each other’s task execution. This was visible when the anesthetic nurse would take over tasks from the circulating nurse with the purpose of helping to create flexibility to enable an appropriate flow during the surgical procedure. This was also visible when the surgeon involved the team members in the surgical technique and the OR nurse was vigilant and aware of the surgeon’s preferences and surgical technique, as shown in this situation [Team 23]:

*The surgical procedure has just begun. The SG says: “We start!” The SG shows the SA how to hold the retractors. The SN works quietly. The SG tells the SA what he sees, what he is doing and why. He speaks softly, so the patient cannot hear him. Together, they talk about the condition of the patient’s knee. The SG describes what he is going to do next. The SN focuses and prepares for what she predicts will be SG's next move or need. The SG drills the nail into the thighbone and says: “I would like to have a…” The SN hands it to him before he has pronounced the name of the instrument. The SG saws the bone. He wants to pull out a nail, but it is stuck. The SN hands the SG an instrument to pull the nail out. Again, the SG talks softly to the SA about the surgical technique. The SN holds the surgical instrument that she predicts is going to be used next; she closely follows the SG movements and action. She is right in her predictions and hands over the instruments without speaking when it is needed by the SG, as though she knows exactly what his next move is going to be.*

Often, these types of teams were found to perform surgery that involved a high degree of complexity, which underlined the benefit of proactive and intuitive coordination and communication when problem solving was needed. This occurred for example in a situation where two OR nurses were preparing for a very complicated surgical procedure, and they had to connect several rarely used surgical instruments and prepare a variety of custom-made replacement materials [Team 29]:

*Suddenly, the SN says: “Oh, these two… they don’t fit together!” The CN thinks and says: “Oh, NO, we have to stop him [the surgeon]. The head [one part of the replacement materials], he has chosen, doesn’t fit in.” She walks quickly to the place where the AN nurse is preparing the patient for the anesthesia and says: “Wait a minute!” Then, she walks in a hurry to the phone and calls the SG. The CN and the SN discuss the size of the different parts of the replacement materials and what to do now. The CN says: “He will come, and he is very annoyed that the person who prescribed the operation was so focused on the thighbone part, when the patient’s acetabulum is so damaged.” They continue talking about which solutions they should opt for. The SG arrives, and together they discuss the possibilities and decide. “We will continue! Never going down on equipment!” SG exclaims.*

Finally, these teams expressed mutual respect: verbally as well as non-verbally, and a remarkable responsibility for the interdisciplinary learning environment in the operating room. This was observed for example when an experienced OR nurse greeted and gave instructions to the surgeon’s assistant in the operating room about the scheduled surgical procedures; and when senior surgeons´ were educating surgical assistants or showing great attention and patience towards newly employed OR nurses.

### Type 2: silent and ordinary communication

In Type 2 teams, interdisciplinary collaboration was guided by shared goals and characterized by mutual respect. Frequently, these teams were performing surgical procedures on patients who underwent standard/routine surgery of short duration which required less exchange of opinions, alignment of expectations, and shared problem solving. Communication and relationship dynamics in these teams were therefore characterized as being more silent and less dynamic than seen in the other types of teams.

This type of silent interpersonal dynamic appeared when the team members performed safe-surgery procedures such as time-out and check-out. Often, the verbal exchange of information in these procedures was very brief without details on the specific surgical procedures, expected challenges, or estimated duration of surgery. Sometimes the execution of the check-out procedure was skipped despite the unit’s safe surgery guidelines.

Another representation of silent communication was visible during the surgical procedures. In these situations, speech acts between team members were informative and instructive, without preceding discussions of uncertainties, expected challenges, or decisions regarding the patient and the surgical procedure, as in this observation [Team 14]:

*The SG picks up the instrument from the table and puts it back again, himself. Unusually, the table is placed between the SG and the SN. Sometimes, the SN hands the instruments to the SG and collects small bone pieces from the SG’s tweezers using a piece of tissue. Occasionally, the SG says what he needs to have. He uses the ball joint reamer [instrument for milling the acetabulum] and says, “54,” to which the SN replies, “Yes” and hands the instrument to the SG. Once more, the SG uses the ball joint reamer and says, “I need a larger number!” He gets the instrument, uses it, and says to CN, “We get a 60!” The CN points to a room outside the operating room and asks the SN, “It is outside, isn’t?” The SN answers: “Yes, and it must be the one without holes!”*

Although the interpersonal dynamics in these teams were often silent during the surgical procedures, a lively conversation was observed between the OR nurses during the preparation for the surgical procedures. Typically, communication between the nurses was focused on the instruments and materials needed, but there was also a lot of small-talk or talk about social life in the unit and about personal issues.

Members of these teams were often familiar with the scheduled surgical procedures and with one another. The routine nature of the surgical procedures influenced the topics of the communication in terms of what was needed to be discussed and arranged. Team members rarely talked about surgical complications, but they always sought to be prepared for the most commonly encountered variations concerning hip and knee replacement procedures and aware of the surgeon’s preferences of instruments [Team 12]:

*The SG takes off his gloves, having just finishing the surgical procedure. The CN says, “Look at these pictures [X-rays]. It is from the next patient! What did we agreed about? What are we going to do?” Then, they talk about which type of hip replacement materials they are going to use for the next patient. They walk together to the closet and look at the different replacement materials and instrument boxes. They make a choice and decide together.*

Team members in these teams were communicating and acting in a manner supportive of shared goals. Goals were not always accurate, clear, or obvious; rather, they were implicit and rarely verbalized. The team members showed awareness of what was important for the task performance, for the patient’s outcome, and for each other’s function. This awareness was expressed in the following [Team 5]:

*The CN says to the SG, “Would you like us to release the tourniquet [decouple the blood pressure cuff] now or do you prefer that we wait a little?” The SG answers, “We wait!” Then, the AN nurse says to the CN, “When you release the tourniquet, please tell me, because I think she is a person [the patient] who could present bradycardia when we release the tourniquet!” “Yes, of course – I will do so!” the CN replies.*

Finally, it was found that familiarity, routine tasks, and knowledge of one another on a personal level established an atmosphere of fellowship and safety, which, occasionally was disturbed by an ironic tone of voice in the operating room. *“This way of speaking together in the operating room is a part of our culture, we are aware of the tone, but sometimes it appears to be too much”* [SN 25].

### Type 3: attentive and ambiguous communication

In Type 3 teams, interdisciplinary collaboration was characterized by health professionals who were guided primarily by functional goals and to a lesser extent by shared goals. Collaboration was characterized by team members expressing disrespect rather than respect, as well as team members using blaming communication rather than problem-solving communication. These teams were found caring for patients who underwent routine as well as complex surgery. Communication and relationship dynamics in these surgical teams were characterized by inattention to one another and by ambiguous speech acts between team members.

Inattentiveness was observed when OR nurses were unprepared to follow the surgeons and their next moves during the surgical procedures, or when it was difficult for OR nurses to get hold of the surgeons prior to surgery, which resulted in prolongation of ongoing surgery or delays of scheduled surgical procedures. The team members’ orientation towards their own goal accomplishment rather than accomplishing shared goal of the team was reflected in their lack of attention and lack of knowledge of what other team members needed to accomplish their specific goals. This was apparent in the variations among team members regarding what was the most effective and efficient way of preparing for surgical procedures [Team 13]:

*The SG enters and completes a very short check-in procedure with the CN and AN nurse. CN says loudly, “We have prepared for a cemented arthroplasty X [she names a specific procedure], and for this procedure we have these materials!” She points to the materials on the table and continues, “Then we have prepared for an uncemented arthroplasty Y [she names another specific procedure] and for this procedure we have these materials!” She points to the materials on another table. The SG replies, “What if it is a Z arthroplasty [he names a third specific procedure], what have you prepared for that procedure?” CN answers, “We haven’t prepared for that procedure, today!” The SG response, “Well, why not? That is too bad!” The CN answers quickly, “You can’t have it!” The SG then comments, “I will stick to my fundamental views on this case about unpacking. In general, I think it is important to think about saving money; we just take the stuff into the operating room and pack it up if we need it.” The CN responds, “Okay, but if it isn’t prepared, you would blame me if we need something during the intraoperative phase!”*

Communication between the health professional in these teams was clearly different from that of the other teams observed. Sometimes communication between team members was inappropriate, and sometimes the tone of voice was ambiguous and disrespectful [Team 34]:

*The team is performing check-in safety procedure. The SN asks, “Antibiotic, is it given?” The AN nurse answers, “No, it has to be given after the biopsy!” The SG adds, “Exactly”. The AN nurse says to the SG, “You’ll tell me when I am allowed to inject the antibiotics, right?” The SG says, “YES, and you will remind me to tell it! It is something one can forget!” The SG continues, “Can I say something regarding the next patient if it is suitable now?” The SN asks, “Yes, but do we have time for the next patient today?” The SG replies, “YES, we do. We are on track! The next patient should not be sedated!” He continues, now very loudly, “Are you listening?” and he follows up by forcefully mentioning the first name of the AN nurse. The AN nurse responds with a single word, “Yes.” After a few minutes, the SG has directed his attention to the SN, who is working by his side connecting the suction line and the surgical coagulator. The SN is struggling with the lines; she is focused because the lines have become tangled together. The SG says very loudly and with an ironic tone of voice, “NO, no, now you have to STOP! You must be true to your own principles! Do you hear? Before, you told me that it doesn’t work to make a Dick Turpin’s knot [a specific way of tying a knot], and now you are standing there tying a double bowline knot – yourself!”*

Several of these teams were working in an atmosphere with a touch of uncertainty, and frequent use of irony and sarcasm was observed, in addition to ambiguous attitudes related to individual team members. These attitudes were sensed when observing a newly employed OR nurse and a senior surgeon collaborating [Team 28]:

*The surgical procedure has just begun. The SN stands on a step stool and she has two instrument tables ahead. She is going to jump down the stool if she has to reach the instruments on the tables behind her. The SG asks, “Do you have a sand pillow?” and the SN answers, “Yes, here!” The SG asks, “Do you have a scissors and a tweezer?” He gets the instruments. The SG asks again, “Then, I must have a tread!” The SN replies with a question, “A lilac?” and the SG answers, “Yes, or a blue one!” The SG continues, “Can I get a chisel?” The SN is searching on the tables in front; she jumps down the stool and searches on the tables behind. The SG is waiting, and after a little while he says loudly, “The nurse can’t find the chisel.” After waiting a little longer, he continues, “The fact that she cannot find it, I view as a sign that she opposes me!” The SN is quiet, and she finds the chisel. The collaboration goes on the same way for minutes. The SG asks, the SN scans the tables and jumps the step stool up and down. Finally, the SG says, “Wouldn’t it be easier if you roll the tables to me?” The SN answers, “I didn’t expect you to use it!” The SG responds, “I always do. ALWAYS!” Now the CN interposes, “Isn’t he nice to you, x?” [She mentioned the first name of SN]. Halfway through the surgical procedure, the SG exclaims loudly, “This is a mess! The conclusion of the surgery today must be: It is fantastic that the surgeon finished at all!” The tense atmosphere continued.*

These teams worked together on surgical procedures of varying degrees of complexity, just as frequently with standard/routine tasks as with advanced/complicated orthopedics surgery. However, when performing very complex surgical procedures, accurate and timely communication was typically observed during the time-out and check-out procedures. When routine surgery was performed, the safe surgery procedures were often poor, inaccurate, or even missing.

### Type 4: highly dynamic and protective exchanges of meaning

In Type 4 teams, interdisciplinary collaboration was characterized by being inconsistent. The interpersonal interactions were highly dynamic in the sense that communication between team members could vary from being respectful, accurate, and problem-solving to being sharp, ironic, disrespectful, and finger pointing. As with Type 2, these teams frequently cared for patients who were undergoing routine surgery of short duration. Communication and relationship dynamics in these surgical teams were characterized by being highly dynamic as a result of contradictions in team members’ cooperation behaviors and personalities.

Contradictions became visible in team members responses to each other, when a sharp and commanding tone was met by silence and short answers, as shown in the following situation [Team 18]:

*The SG and the SA are trying to replace the leg but it doesn’t work out. The SG exclaims loudly, “No, dammit, the monkey hand [nickname for a certain instrument], NOW!” The SG takes the offered instrument and manipulates the leg, and it snaps into place. The SG says, “Minus 4 [size of the hip material]!” and the SN finds it. Together, they check the size, and the SG responds in a sarcastic tone, “THANK YOU!” The SN is quiet and focused on her tables and the instruments. Beyond the exchange of words regarding the instruments, there was no communication between the SN and SG. At the end of the surgical procedures, the SN asks the SG, “Should I fill out the paperwork, or is it something you do?” The SG answers shortly: “Something I do!”*

Additionally, this type of contradictory and highly dynamic communication was observed when disrespectful behavior and finger-pointing attitudes were met by collaborators who responded in a respectful and problem-solving manner.

Occasionally, it was difficult to determine whether the participant expressed mutual respect or not because of the ironic and teasing tone of voice. An atmosphere of insecurity could sometimes be sensed during the surgical procedures and was clarified in interviews conducted after the observation. The health professionals had developed different strategies to manage tense or strained situations with their colleagues in the operating room. Some choose silence and focused on their tasks*.* For example, [SG 1] *“I freeze the situation or kill the discussion by not attending”* or [SN 5]: “*I keep my mouth shut.”* Others confronted the tough tone [SN 5]: “*I would tell the person that my limits are exceeded, or I would say I have a sense that you are a little annoyed today, what is it about?”* Others took a problem-solving approach, for example, [AN Nurse14]: “*Someone yells and shouts about how bad things are going. Perhaps I have been there before myself. Now, I am saying maybe it isn’t well-functioning, but you should move back, take it easy, and try to talk about it together.”*

Episodes of disrespectful behaviour were also observed in these teams, reflected in several ways, as team members showed minor temper tantrum, used disrespectful language, argued in a commanding tone, or humiliated other team members by shaming them for being unprepared or unfocused. Such episodes of disrespectful behaviour created a tense atmosphere for collaboration, as expressed by an OR nurse immediately after a surgical procedure where this kind of behaviour was observed [OP 33]:

*“I like that we constantly have dialogue about what is going to happen! For the most part, we are good at the planning part. But there are just some combinations that do not work quite well! And it marks you immediately. It does! In reality, it depends on individuals. And one can also notice that there are some surgeons and some OR nurses that doesn’t fit together! Then, the surgeon is right up in the red zone already before we start, and it spills over! I don’t like it at all. In my opinion, it is unprofessional of all parties involved, and it provides a very annoying mood all day. It might be hard, to be in for a full day. Because the room will explode if you say just one wrong word, or people jump down the throats of each other if something is upside down. In these situations, I am aware to not do anything wrong, since I know that the whole thing will explode.”*

Finally, health professionals in these teams often talked about topics that were irrelevant for the surgical procedures. In some cases, these conversations served as invitations to newcomers to participate in the community of the surgical teams. In other cases, the conversations between individual team members were of a nature that excluded other team members, who then became quiet [Team 16].

*The SG asks the CN if she has got a new haircut. She answers, “Yes, and haven’t you lost weight?” The SG replies: “Yes, I am going to complete a marathon, so I must.” The newly employed SN, the SA, and the AN nurse are quiet and focus on their tasks. The conversation about running continues, while they work with the surgical procedure and the CN quit by saying to SG: “You have also so much confidence and charm!”*

However, these Type 4 teams were typically observed performing routine surgery, so solutions to instrumental or surgical challenges were rarely required.

## Discussion

The purpose of this ethnographic study was to explore communication and relationships in interdisciplinary surgical teams at the micro level in contexts of variable complexity using the theory of relational coordination. Four different patterns of communication and relationship in interdisciplinary surgical teams were identified; and the study may indicate a connection between these patterns and the level of complexity in surgery. Together, these results provide important insight concerning interpersonal dynamics, teamwork, and performance within interdisciplinary surgical teams in operating rooms.

The identification of different communication and relationship patterns enables a nuanced interpretation of the interdisciplinary collaboration between team members in surgical teams. The relationships were found to be not only role-based, but in fact both role-based and person-based. Role-based relationships are consistent with Gittell’s [25] theory of relational coordination describing appropriate and inappropriate communication and relationship dynamics; which must be considered to be given, since these perspectives were used as the theoretical framework in the qualitative content analysis provided. The person-based relationships emerged, when team members expressed respect not only for their interdisciplinary colleagues’ skills and professionalism, but also for each other as unique individuals (Type 1); or when team members used a familiar tone of voice during surgical procedures (Type 2). Other types of person-based relationships were visible, when team members were inattentive to each other (Type 3), or when disrespect was ignored and responded to with silence (Type 4). The findings that communication and relationship dynamics in interdisciplinary surgical teams are both role-based and person-based adds nuances to the theory of relational coordination. Gittell [36] had previously argued for the possibility of extending the relational coordination theory to account for personal relationships. However, it has never been demonstrated before. In order to further explore the character of relationships between surgeons, anesthesiologists, and nurses collaborating at the micro level, further studies on the current topic are recommended.

Observing collaboration in interdisciplinary surgical teams enabled the identification of different communication and relationship patterns, reflecting what communication and coordination in surgical teams looked like, when it succeeded, and when it was not successfully achieved. The different patterns can be interpreted as reflections of appropriate and inappropriate interpersonal team dynamics in surgical teams. According to Vincent et al. [38], it is crucial to study errors but also to study teamwork and how threats to patient safety are successfully managed within interdisciplinary collaboration in surgical teams. It might be possible to learn more about threats to patient safety from the findings in this study. Proactive and intuitive communication patterns, as seen in surgical teams of Type 1, might contribute positively to patient safety culture in the operating room and might influence both surgical performance and patient safety. Therefore, learning from these teams might improve efficiency and effectiveness in surgical teams and may enhance quality treatment and patient outcomes. Furthermore, it might be essential to prevent inappropriate dynamics, inaccurate, and disrespectful communication patterns, as seen in surgical teams as Type 3, which expressed inattentive and ambiguous ways of communicating. In order to improve collaboration, safety culture, and quality of treatment, this ethnographic study suggests future team training to promote proactive and intuitive communication patterns, and to prevent inattentive and ambiguous communication patterns.

In the teams characterized by a proactive and intuitive pattern of communication (Type 1), there was an explicit effort to know what was going on around oneself in the operating room. This awareness was considered to be very crucial knowledge in order to perform surgical procedures of high complexity, which was frequently the case for these teams. This essential ability might be comparable with the category labelled *situation awareness* in the concept of non-technical skills identified in several studies focusing on health professionals in the operating room [3, 5, 7–11, 39–41]. The communication and relationship patterns identified in the present study can be interpreted as descriptions of appropriate and inappropriate interpersonal team dynamics in surgical teams. Descriptions about how different communication and relationships dynamics show up and how individual team members manage to be a part of teamwork; skills that may be associated with categories as *Decision-making, Communication, Teamwork,* and *Leadership,* also contained in the non-technical skills concept. Therefore, it may be proposed that observation of relational coordination in surgical teams can produce valuable insight regarding how teamwork can be improved at the micro level, as well as how health professionals´ non-technical skills in the operating room can be strengthened. Further work is required to develop a tool for behavioral observation markers of interpersonal dynamics in surgical teams based on the dimensions found in the theory of relational coordination.

Collaboration in interdisciplinary surgical teams was found to be situated in a very complex and changing clinical practice. Health professionals expressed that the quality and effectiveness of performance was challenged by frequent changes in the surgical schedule and interdependence among members of the surgical teams. These findings support previous research, which found that teamwork in interdisciplinary surgical teams is seriously challenged by interdependence, time constraints, and uncertainty [4, 16, 20, 42]. In this study, health professionals described the uncertainties with ambivalent feelings. On the one hand uncertainties gave rise to job satisfaction on the other hand uncertainties were the source of frustrations. There might be similarities between these ambivalent feelings expressed by health professionals in this study and the span between challenges and protections described in an ethnographic study focusing on operating room nurses [20]. Sørensen described the span between “getting a kick out of the uncertainties” and “being stuck in the routine.” It might be assumed that high changeability can be both conducive and inhibitory to the development of appropriate interdisciplinary collaboration. Surgical teams, using communication and relationship patterns such as Type 1 and Type 2, might have established relationships between team members that enabled solutions in changing situations to be found and frustrations to be prevented. While surgical teams, using communication and relationship patterns such as Type 3 and Type 4, might have more unsustainable relationships that challenged problem solving in changing situations and fueled underlying frustrations. Additional studies will be needed to develop a full picture of how different levels of uncertainties, interdependency, and time constraints influence communication and relationship dynamics in surgical teams at the micro level.

In line with previous studies [23, 42–44], the findings indicate that there might be associations between health professionals’ experiences of mutual respect and trusting one another, and the communication and relationship patterns in surgical teams. Edmondson [44] highlighted the need for trust and respect for surgical teams to improve their quality and effectiveness, and the need for anticipating the dangerous silence which may reflect an inappropriate safety climate [45]. Unfolding these needs, Edmondson [44] used the concept psychological safety to describe a team climate in which team members trust each other and feel safe to express concerns, disagreements, and feelings. However, this study showed that it might be challenging to establish psychological safety in the surgical teams in operating room when interpersonal dynamics overruled the scene, and inappropriate or even disrespectful communication patterns dominated the operating room. These findings are consistent with concerns adduced by Leape et al. [46], when they concluded that disrespectful behavior posed a threat to patient safety and undermined collaboration in surgical teams. According to Leape et al., creating a culture of respect in health care is needed to secure patient safety and foster an appropriate culture of safety [46, 47]. This seems to be an essential issue also for improving relational coordination at the micro level between health professionals in surgical teams in the operating room, and point to an important area for future research.

In this study, health professionals expressed the need for exchange of reflections and debriefing several times. However, initiatives to conduct such meetings in the surgical team were never observed. This request for exchange of reflections and debriefing in surgical teams seems to be in consistent with recent studies [42, 48]. Nawaz [42] emphasized the importance of intercollegial feedback and interdisciplinary reflections for surgical team to improve efficacy and learn from experiences to secure patients safety. However, for successful feedback and learning in the operating room, all health professionals need to be open-minded to criticism and be responsive to one another. Though intercollegial feedback and debriefing were absent during the surgical procedures observed in this study, exchange of reflections after surgical procedures and learning from experiences were practiced between newly employed OR nurses and experienced OR nurses and between AN nurses. It is encouraging to compare these learning activities with that concept of *Learning Cycle in Orthopedics* presented by Nawaz et al. [42], who identified four steps in a cyclical learning process: *Diagnose*, *Design*, *Act*, and *Reflect*. This might be a useful model for use in surgical teams in order to improve learning environment and teaming, and it might be especially profitable for surgical teams that exhibited *inattentive and ambiguous communication* patterns (Type 3) or surgical teams that exhibited *contradictory and highly dynamic communication* (Type 4). However, a systematic use of feedback and learning processes in the operating room might foster opportunities for improvement of mutual trust in all types of surgical team, as well as enhancement of treatment and care of patients. Nawaz et al. proposed [42] that surgeons should facilitate such learning processes and undertake the appropriate leadership roles for successful implementation of intercollegial feedback in surgical teams. There is room for further progress in identifying structural, relational, or work process interventions supporting a learning feedback culture in surgical teams.

In this observational study, the interdisciplinary surgical teams that exhibited proactive and intuitive communication (Type 1) were using appropriate coordination strategies, by connecting team members to one another though a broad acceptance of shared goals, shared knowledge, mutual respect, and by timely, accurate, and problem-solving communication. Whereas interdisciplinary surgical teams exhibiting inattentive and ambiguous communication (Type 3) were using more inappropriate coordination strategies. These findings support previous studies on this particular clinical context focusing on adaptive coordination strategies [19] and adaptive capacity [20] in surgical teams. Nevertheless, the comparison also shows significant differences. Where Bogdanovic [19] describes adaptive strategies based on semi-structured interviews of health professionals; the present study describes communication and coordination based on observation of behavior and semi-structured interviews with health professionals. This may provide a significant qualitative difference, given that there may be differences between what the health professionals say they do; and what they actually do in their daily task performance. The adaptive coordination strategies lying beneath surgical teams task-management presented by Bogdanovic (planning, task distribution, prioritization, delegation, clarification of task, team and process monitoring and assistance) [19] could be consistent with the coordination strategies used by surgical teams characterized as having proactive and intuitive communication (Type 1) observed in the present study. It might be that team members in teams characterized by having inattentive and ambiguous communication (Type 3) also wish to be guided by appropriate adaptive coordination strategies but were disrupted and disturbed due to inappropriate interpersonal relationships and lack of mutual respect.

Findings from the present study shows that the four distinct patterns of communication and relationships occur with unequal frequency in surgical procedures with low and high level of complexity. Surgical procedures with a high level of complexity were performed by surgical teams with communication and relationship patterns of Type 1 or Type 3 in 11 out of 15 procedures. Surgical procedures with a low level of complexity were performed equally by all teams; but teams with communication and relationship patterns as seen in Type 2 and Type 4 were performing surgical procedures with a low complexity in 10 out of 13 procedures. It is therefore likely that such connections exist between communication and relationship dynamic and the level of complexity in surgical procedures; but it is from an epistemological point of view beyond the purpose of this ethnographic study to examine correlation between relational coordination and level of complexity.

### Limitations

There are several limitations to these findings. Firstly, data derived from observations in two highly specialized orthopedic surgery units performing hip- and knee replacement operations, with the same group of surgeons, the same management team, and in the same university hospital; but in two geographically different places and with different levels of complexity. A higher degree of diversity and a stronger generalizability might have been taken into account with a multi-case study design. However, the extension of observations of interdisciplinary team work in few selected operating rooms facilitated an in-depth study containing a very rich amount of data, which could have been very hard to achieve if several surgical units were involved [28]. Even though knowledge from one single case-study cannot be formally generalized, it can enter into the collective process of knowledge accumulation in a given field and thereby be valuable [49–51].

Secondly, in any ethnographic study the relationship between the participants being observed and the observer is crucial. To ensure the rigor of the qualitative inquiry, reflections were needed to address a potential concern that team members were communicating and acting in artificial ways due to the observer being present in the operation room. According to Hammersley and Atkinson [29], ethnographic principles are used in practice by studying interactions between participants in their everyday context. In this study, the first author was present in each of the surgical units for 25 days during a period of 4 months; and team members were observed during three to 10 surgical procedures with a duration of a half to nearly 6 hours. It is likely impossible, to act or communicate in an artificial or imagined way in the collaboration with colleagues during surgical procedures for such a long period.

Thirdly, the deductive approach in the coding phase of the content analysis presents some challenges. The researchers could “be seduced by the theory” and thereby be more focused on capturing and interpreting the perspectives in the direction of the predetermined theoretical concepts, with the risk that new or critical perspectives are overlooked or neglected and therefore not intercepted. To overcome some of those limitations related to neutrality and trustworthiness a coding scheme has been developed and discussed between the researchers, and a variety of fieldnotes and participant quotations concerning communication and relationships have been presented. Additional data and perspectives, deriving from interviews as well as focus group interviews with the health professionals, were analyzed and presented in the more extensive dissertation [52]. These perspectives were focusing on other characteristics of the interdisciplinary surgical team work, such as interdependency, uncertainty, and time pressure in the operating room.

Finally, this study was limited to surgical units in the public health care system in Denmark. Further cross-cultural research is needed to explore the transferability to other clinical and cultural contexts.

## Conclusion

Health professionals in surgical teams perform surgical procedures in a context of variable complexity, characterized by frequent changes and uncertainties in the daily surgical program, a high degree of interdependency among team members, and a strong focus on time and resource consumption. This study is complementary to previous studies on relational coordination in surgical units, because the ethnographic study has created opportunities to discover relational coordination between health professionals at the micro level. Exploring communication and relationship patterns between health professionals in interdisciplinary surgical teams at a micro level facilitated a differentiated dynamic picture of teamwork quality rather than a static snapshot offered by measurements showing different levels of relational coordination at the team level. The present study contributes additional knowledge by using ethnographic principles in participant observations as a method for exploring communication and relationships between surgical team members in their daily task performance and thereby identifying four different communication and relationship dynamics in contexts of variable complexity. Relational coordination in surgical teams was revealed to be both role-based as well as based on personal relationships established through intersubjective work experience between team members over time. As a result, the argument that relational coordination is only about role-based relationship has been challenged.

## Data Availability

Further information about the data used and analyzed during the study may be available from the corresponding author on reasonable request.

## Abbreviations

AN nurse: Anesthetic nurses
AN: Anesthesiologist
ASA: The American Society of Anesthesiologists
CN: Circulating nurse
NA: Nurse assistant
OR nurse: Operating room nurse
SA: Surgical assistant
SG: Surgeon
SN: Surgical nurse
